# NLRP3 inflammasome in endothelial dysfunction

**DOI:** 10.1038/s41419-020-02985-x

**Published:** 2020-09-18

**Authors:** Baochen Bai, Yanyan Yang, Qi Wang, Min Li, Chao Tian, Yan Liu, Lynn Htet Htet Aung, Pei-feng Li, Tao Yu, Xian-ming Chu

**Affiliations:** 1 Department of Cardiology, The Affiliated Hospital of Qingdao University, Qingdao, 266000 China; 2 Department of lmmunology, School of Basic Medicine, Qingdao University, Qingdao, 266071 China; 3 Institute for Translational Medicine, The Affiliated Hospital of Qingdao University, Qingdao, 266021 China; 4 Department of Cardiac Ultrasound, The Affiliated hospital of Qingdao University, Qingdao, 266000 China; 5 Department of Cardiology, The Affiliated Cardiovascular Hospital of Qingdao University, Qingdao, 266032 China

**Keywords:** Cell biology, Cardiovascular diseases

## Abstract

Inflammasomes are a class of cytosolic protein complexes. They act as cytosolic innate immune signal receptors to sense pathogens and initiate inflammatory responses under physiological and pathological conditions. The NLR-family pyrin domain-containing protein 3 (NLRP3) inflammasome is the most characteristic multimeric protein complex. Its activation triggers the cleavage of pro-interleukin (IL)-1β and pro-IL-18, which are mediated by caspase-1, and secretes mature forms of these mediators from cells to promote the further inflammatory process and oxidative stress. Simultaneously, cells undergo pro-inflammatory programmed cell death, termed pyroptosis. The danger signals for activating NLRP3 inflammasome are very extensive, especially reactive oxygen species (ROS), which act as an intermediate trigger to activate NLRP3 inflammasome, exacerbating subsequent inflammatory cascades and cell damage. Vascular endothelium at the site of inflammation is actively involved in the regulation of inflammation progression with important implications for cardiovascular homeostasis as a dynamically adaptable interface. Endothelial dysfunction is a hallmark and predictor for cardiovascular ailments or adverse cardiovascular events, such as coronary artery disease, diabetes mellitus, hypertension, and hypercholesterolemia. The loss of proper endothelial function may lead to tissue swelling, chronic inflammation, and the formation of thrombi. As such, elimination of endothelial cell inflammation or activation is of clinical relevance. In this review, we provided a comprehensive perspective on the pivotal role of NLRP3 inflammasome activation in aggravating oxidative stress and endothelial dysfunction and the possible underlying mechanisms. Furthermore, we highlighted the contribution of noncoding RNAs to NLRP3 inflammasome activation-associated endothelial dysfunction, and outlined potential clinical drugs targeting NLRP3 inflammasome involved in endothelial dysfunction. Collectively, this summary provides recent developments and perspectives on how NLRP3 inflammasome interferes with endothelial dysfunction and the potential research value of NLRP3 inflammasome as a potential mediator of endothelial dysfunction.

## Facts

- NLRP3 inflammasome is involved in a wide range of pathological conditions and diseases as intracellular innate immune sensors.
- NLRP3 inflammasome-mediated inflammation and pyroptosis play a pivotal role in endothelial dysfunction.
- Noncoding RNA, as an emerging disease biomarker, may regulate endothelial function by mediating the NLRP3 inflammasome signaling pathway.
- Certain drugs, such as statins, hypoglycemic agents, and other anti-inflammatory or antioxidant drugs, can improve vascular dysfunction by inhibiting the NLRP3 inflammasome signaling pathway.

## Open questions

- What is the molecular mechanism underlying NLRP3 inflammasome-related endothelial dysfunction?
- How does NLRP3 inflammation perceive information about different mediators?
- Whether different agonists affect endothelial function through a single cascade of events or through distinct pathways to activate NLRP3 inflammation?
- Do other inflammasomes such as NLRP1, NLRC4, NLRP6, AIM2, noncanonical inflammasomes, and upstream pathways affect endothelial function?

## Introduction

The innate immune system is the primary mechanism by which most organisms respond immediately to infections or injury. Pattern-recognition receptors (PRRs) in the host are activated, which recognize molecules released by pathogens or damaged cells. These molecular signals are known as pathogen-associated molecular patterns (PAMPs) and damage-associated molecular patterns (DAMPs)^1^. The PRR family has many members, including Toll-like receptors (TLRs), NOD-like receptors (NLRs), C-type lectin receptors, retinoic acid-inducible gene (RIG)-I-like receptors (RLRs), as well as several intracellular DNA sensors^2^. Innate immune cells play critical roles in PRR-initiated innate inflammatory response, but the effect of nonimmune cells, such as endothelial cells (ECs), is also a force to be reckoned with^3^. The transcriptional upregulation of pro-inflammatory genes by PRRs triggers a cascade of inflammatory responses. Although inflammation has the beneficial effect of limiting cellular and organ damage, disruption in its regulation may result in a sustained inflammatory response and ultimately, local or systemic inflammation^4^.

The inflammasome, a type of PRR, constitutes an essential component of the innate immunity^5^. Abnormal activation of inflammasomes is the pathogenesis of various inflammatory diseases^6^. The inflammasome is a high-molecular-weight protein complex that acts as a cytosolic innate immune signaling receptor that senses PAMPs or DAMPs and mediates a highly inflammatory state. The first inflammasome was discovered in 2002^5^ following which, various inflammasomes have been identified, including NLR-family pyrin domain-containing protein (NLRP) 1, NLRP3, NLRP6, NLR-family caspase recruitment domain (CARD)-containing protein 4 (NLRC4), absent in melanoma 2 (AIM2), and pyrin inflammasomes^7^. Among them, the NLRP3 inflammasome is the most well-characterized, largest multimeric protein complex to date.

Microvascular ECs at a site of inflammation are both active participants and regulators of inflammatory processes. During the transition from acute inflammation to chronic inflammation or from innate immunity to adaptive immunity, the characteristics of ECs change through EC activation, rapid recruitment of neutrophils, and increased vascular leakage of plasma proteins. These changes eventually lead to endothelial dysfunction^8^. Interleukin (IL)-1β is an important pro-inflammatory cytokine released during the endothelial inflammatory response^9^. Interestingly, activation of NLRP3 inflammasome can produce high numbers of IL-1β.

Additionally, high-quality research has shown that NLRP3 inflammasome activation is correlated with multiple chronic inflammatory diseases and metabolic disorders, including obesity, hypertension, diabetes, atherosclerosis, neuroinflammation, retinopathy, stroke, and cancer^10–16^. These diseases also have a close connection with the dysregulation of the endothelium^17^. These studies indicate that the activation of NLRP3 inflammasome in ECs under pathophysiological conditions may aggravate endothelial dysfunction, leading to various diseases. In recent years, endothelial function research has increasingly focused on the regulatory role of NLRP3 inflammasome. In this review, we attempt to summarize the latest progress and development trends in the NLRP3 inflammasome research, highlighting its role in redox regulation and endothelial dysfunction. Also, we emphasize the current knowledge of the factors that mediate and influence NLRP3-related endothelial dysfunction and potential therapeutic targets, which may be helpful for the treatment of endothelial dysfunction-related diseases.

## Inflammasome family

**Fig. 1:**
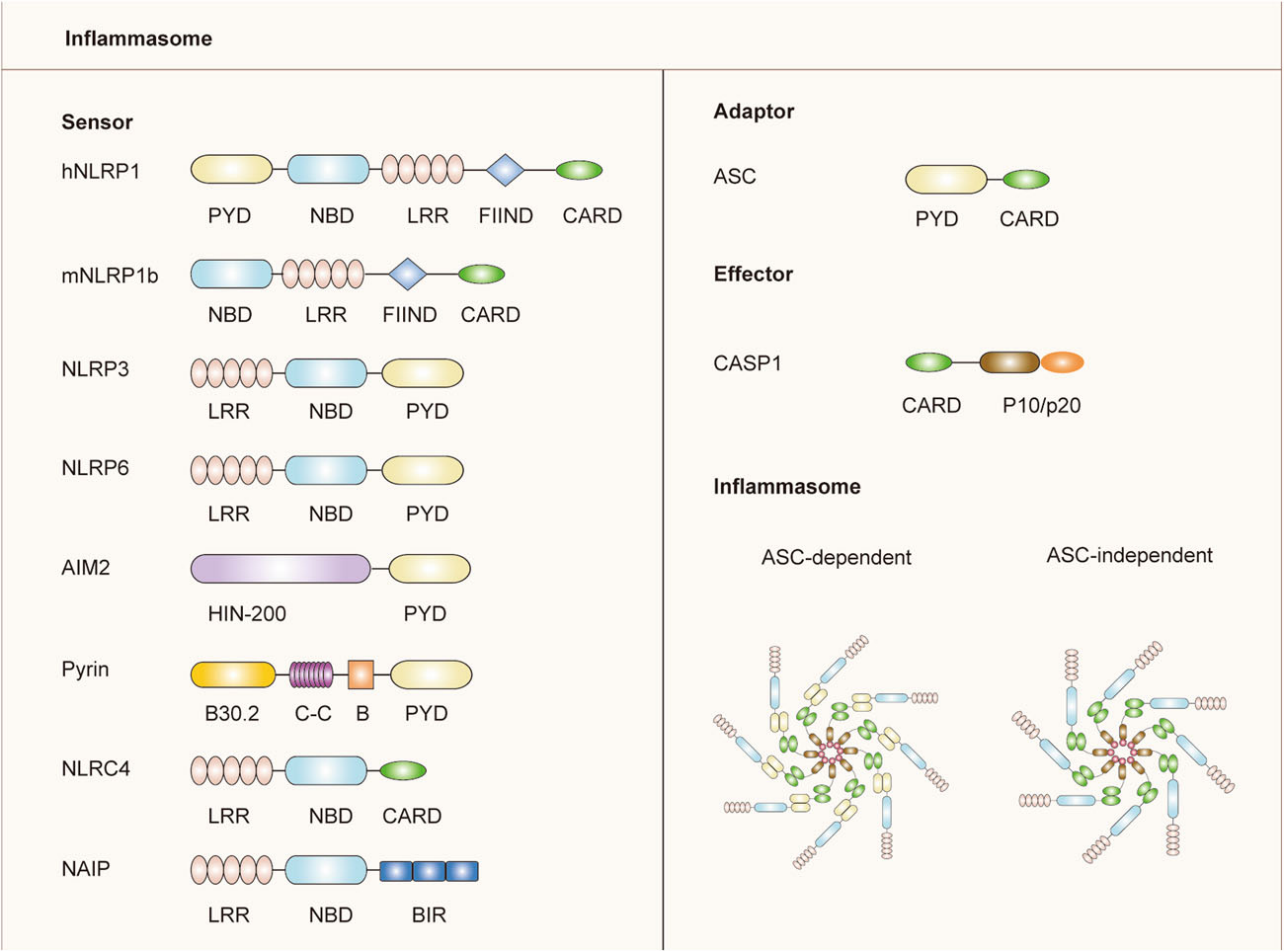
Domain structure of representative inflammasome. Inflammasome is a protein complex formed by the aggregation of inflammasome sensor, adaptor protein ASC, and effector protein caspase-1. PYD pyrin domain, NBD nucleotide-binding domain, LRR leucine-rich-repeat domain, FIIND function-to-find domain, CARD caspase activation and recruitment domain, C–C coiled-coil domain, B B-box domain, BIR baculovirus inhibitor of apoptosis repeat, ASC apoptosis-associated speck-like protein containing a CARD, CASP1 caspase-1.

**Fig. 2:**
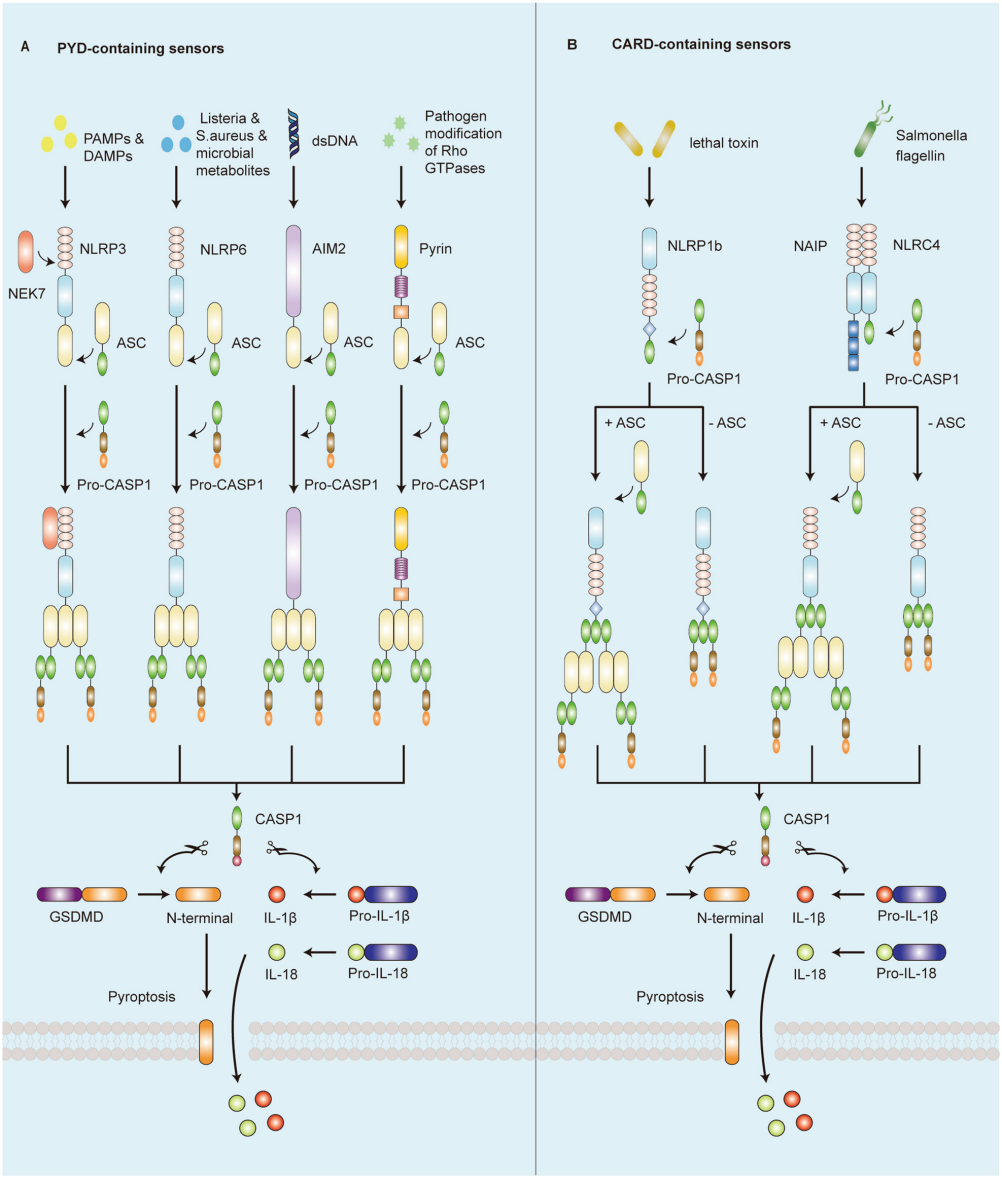
The canonical inflammasome activation pathway occurs by sensing diverse pathogen-associated molecular patterns (PAMPs) and damage-associated molecular patterns (DAMPs). **a** NLRP3, NLRP6, AIM2, and pyrin, containing a PYD, binds to the PYD of ASC, allowing the ASC to activate caspase-1 by interacting with the CARD of pro-CASP1. Activated caspase-1 triggers the cleavage of pro-interleukin (IL)-1β, pro-IL-18, and gasdermin D (GSDMD), and then releases the N-terminal domain of GSDMD to induce pyroptosis, followed by the release of IL-1β and IL-18. **b** NLRP1b and NLRC4, containing a CARD, activate caspase-1 by directly binding the CARD of pro-caspase-1 without ASC or binding the paired ASC scaffold. The presence of ASC can enhance the assembly of the sensor protein containing a CARD and the activation of caspase-1. The molecular process after caspase-1 activation is the same as part A.

The inflammasome is a molecular platform that drives effector caspase-1 activation, which is assembled by NLRs, AIM2-like receptors (ALRs), or pyrin that can directly or indirectly (via the adaptor apoptosis-associated speck-like protein containing a CARD (ASC)), activate caspase-1 (Fig. 1)^18^. Structurally, the N-terminal domain of these sensors includes a CARD or a pyrin domain (PYD). The adaptor ASC is composed of a PYD and a CARD, while caspase-1 contains a CARD^19^. Recently, studies have elucidated that NLRP1, NLRP3, NLRP6, AIM2, and pyrin carry a PYD in their N-terminal region, whereas NLRP1b and NLRC4 contain a CARD. The sensor protein, containing a PYD, binds to the PYD of ASC, allowing the ASC to activate caspase-1 by interacting with the CARD of pro-caspase-1. In contrast, a CARD-containing sensor protein may activate caspase-1 by directly binding to the CARD of pro-caspase-1 without ASC^20^. However, the presence of ASC can enhance the assembly of the sensor protein containing a CARD and the activation of caspase-1 (Fig. 2)^21^.

Furthermore, different sensor proteins respond to different activators (Fig. 2). NLRP1 inflammasome is activated by specific pathogens, such as anthrax lethal toxin, muramyl dipeptide (MDP), *Toxoplasma gondii*, *Shigella flexneri*, and *Listeria monocytogenes*, which cause proteolysis of the NLRP1 N terminal^21,22^. NLRC4 inflammasome can be activated by bacterial ligands, including flagellin and components of the type III secretion system (T3SS), which are sensed and bound by NOD-like receptor family apoptosis-inhibitory protein (NAIP) to induce NLRP4 oligomerization^23^. NLRP6, one of the newest and less-researched members of the NLR family, is overexpressed explicitly in human and mouse intestinal epithelial cells. Microbial metabolites secreted by intestinal commensal bacteria can modulate NLRP6 activity and IL-18 secretion^24^. Moreover, certain pathogens, such as *Listeria monocytogenes*, *Salmonella typhimurium*, and *Staphylococcus aureus*, have been shown to activate the NLRP6 inflammasome as part of the host defense^25^. AIM2 acts as a cytoplasmic sensor that can detect and directly bind to double-stranded (ds) DNA of any sequence produced by pathogens or cellular perturbations, inducing the capability of self-oligomerization and then recruiting ASC for activation of inflammasome^26^. In 2004, pyrin was first shown to self-assemble into an inflammasome by recognizing the inactivation of RhoA GTPase, a molecular switch that controls the dynamics of the cytoskeleton^27^. Recently, studies found that bacterial toxins can modify the Switch-I region of RhoA and trigger pyrin activation, such as TcdA and TcdB secreted from *Clostridium difficile*^27^. Additionally, a most recent study demonstrated that bile acid analogs of microbial origin, such as BAA473 and BAA485, can also activate the pyrin inflammasome^28^. NLRP3 inflammasome is triggered by numerous stimuli, including endogenous molecules, crystalline substances, pathogenic microbes, and ATP. NLRP3 inflammasome functions as a signal integrator to sense several cellular signals, including ion fluxes such as potassium (K^+^) efflux and calcium (Ca^2+^) influx, lysosomal leakage, mitochondrial dysfunction, and ROS production^29^.

Overall, different inflammasomes play unique roles in the defense against specific pathogens. Overactivation of inflammasomes can lead to significant inflammatory responses and pathological changes that are closely related to autoimmunity and autoinflammation. Therefore, inflammasomes are involved in a wide range of pathological conditions and diseases as intracellular innate immune sensors for conditions like infection or aseptic inflammation, metabolic syndrome, age-related chronic inflammatory disease, and autoimmune disease^7,10^.

## NLRP3 inflammasome activation

NLRP3 inflammasome contains innate immune sensor NLRP3, adaptor molecule ASC, and effector protease pro-caspase-1^30^. The activation of NLRP3 inflammasome is essentially the autocatalytic activation of caspase-1. Once activated, NLRP3 acts as a sensor molecule that occurs in self-oligomerization and recruits ASC via homotypic PYD–PYD interaction, which induces the assembly of ASC into large speck-like structures. Subsequently, aggregated ASC recruits pro-caspase-1 via CARD–CARD contact, leading to autocatalytic activation of caspase-1. The function of activated caspase-1 heterotetramers is proteolytic activation of the pro-inflammatory cytokines IL-1β and IL-18, and of a soluble cytosolic protein gasdermin D (GSDMD). Upon proteolysis, the oligomerized gasdermin N can bind membrane lipids and form membrane pores to mediate the nonconventional secretion of IL-1β and IL-18. In parallel, cells undergo a pro-inflammatory type of cell death known as pyroptosis^17,31,32^.

**Fig. 3:**
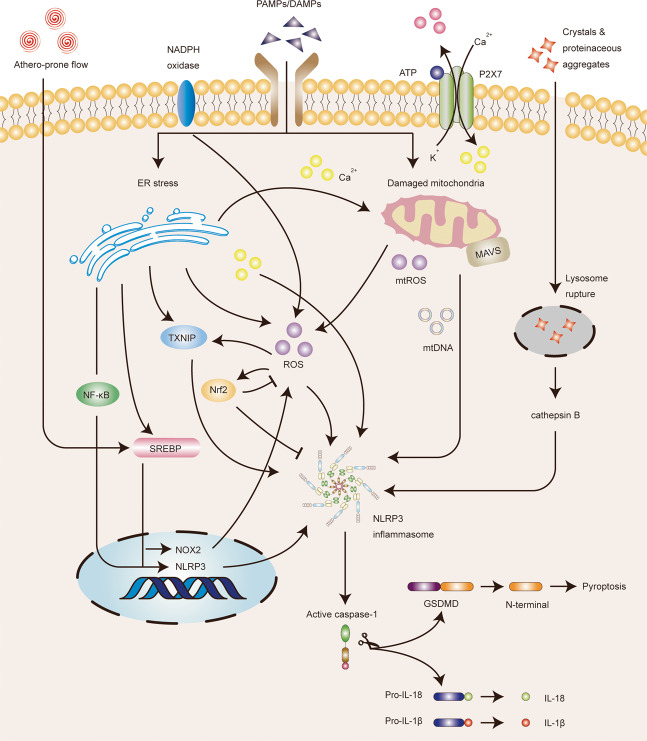
Reactive oxygen species (ROS) contribute to the NLRP3 inflammasome activation in endothelial cells. A wide range of pathogen-associated molecular patterns (PAMPs) or damage-associated molecular patterns (DAMPs) trigger NLRP3 inflammasome activation by inducing potassium (K^+^) efflux, calcium (Ca^2+^) influx, lysosomal leakage, mitochondrial dysfunction, and ROS production. ROS mainly derived from endoplasmic reticulum (ER) stress, damaged mitochondria, and NADPH oxidase. ER stress could activate the NF-κB, TXNIP, and SREBP signaling pathways, Ca^2+^ release, and ROS production. Moreover, athero-prone flow can also mediate SREBP signaling pathways. The release of mtROS and mtDNA in damaged mitochondria might activate NLRP3 inflammasome. Furthermore, mitochondrial antiviral signaling protein (MAVS) is capable of molulating the recruitment and localization of NLRP3. However, NF-E2-related factor 2 (Nrf2) activated under ROS-induced stress conditions can inhibit NLRP3 inflammasome activation. Taken together, ROS is an intermediate factor involved in multiple signaling pathways and can trigger the activation of NLRP3 inflammasome.

Multiple cellular signals are thought to account for NLRP3 inflammasome activation, including ion fluxes such as K^+^ efflux, Cl^−^ efflux, Ca^2+^ influx, and Na^+^ influx, lysosomal leakage, mitochondrial dysfunction, and ROS production^33^. These various upstream signaling pathways may be interrelated or independent (Fig. 3). Additionally, noncanonical NLRP3 inflammasome activation and alternative NLRP3 inflammasome activation are two other forms of activation^34^. Noncanonical NLRP3 inflammasome activation is induced by cytosolic LPS that is sensed by noncanonical caspases 4/5 in humans and caspase-11 in mice. Subsequently, caspases 4/5/11 are activated to cleave GSDMD, resulting in K^+^ efflux and pyroptosis^35^. Alternative NLRP3 inflammasome activation is species-specific, as it only occurs in human and porcine monocytes. This activation is induced by TLR4–TIR-domain-containing adaptor-inducing interferon-β (TRIF)–receptor-interacting serine/threonine-protein kinase 1 (RIPK1)–Fas-associated protein with death domain (FADD)–CASP8 signaling pathway. Of note, this pathway does not depend on K^+^ efflux and does not induce ASC speck formation and pyroptosis^36^.

Among the aforementioned multiple activation mechanisms, ROS bridges the interaction between NLRP3 inflammasome and endothelial dysfunction. Specifically, ROS are one of the first intermediates produced by many known NLRP3 inflammasome activators, and are involved in the mechanisms that trigger the formation and activation of the NLRP3 inflammasome, thereby promoting tissue inflammation and activating immune response^37^. Thioredoxin-interacting protein (TXNIP), nuclear factor-kappaB (NF-κB), and the transcription factor nuclear factor erythroid 2-related factor 2 (Nrf2) are proteins involved in the response to oxidative stress, which links ROS to NLRP3 activation (Fig. 3)^38^. Moreover, aberrant production of ROS may increase nitric oxide (NO) catabolism, leading to a decrease in NO bioavailability. This imbalance in NO–ROS leads to the expression of inflammation-related genes and upregulation of inflammatory proteins, which in turn destroys endothelial function^39^.

ROS production in the mitochondria is the primary source of cellular ROS. Indeed, mitochondrial ROS (mtROS) are involved in NLRP3 inflammasome activation (Fig. 3). The endothelial mitochondria act as critical signaling organelles that play a crucial role in endothelial function, including subcellular location, dynamics, biogenesis, mitophagy, autophagy, ROS production, calcium homeostasis, regulation of EC death, and heme synthesis^40^. Intriguingly, the accumulation of damaged mitochondria has also been proposed to be essential for NLRP3 activation. In addition to increased mtROS, the exposure of mtDAMPs, such as mtDNA and cardiolipin to the cytosol, alterations in metabolite levels, and mitochondrial antiviral signaling protein, can also contribute to NLRP3 activation^38^.

ROS also bridge the interaction between endoplasmic reticulum (ER) stress and NLRP3 inflammasome activation (Fig. 3). ER stress has been proven to activate NLRP3 inflammasome and promote the development of endothelial dysfunction. Specifically, ER stress-induced protein misfolding, imbalance of sterol synthesis and distribution, and release of Ca^2+^ and ROS can trigger NLRP3 inflammasome activation^41^. Meanwhile, multiple molecular pathways during severe ER stress are also involved in the activation of the NLRP3 inflammasome, including p38 mitogen-activated protein kinase (MAPK) pathway, Jun-N-terminal kinase (JNK) signaling, X-box-binding protein-1 (XBP1), CCAAT/enhancer-binding protein–homologous protein (CHOP), NF-κB, and TXNIP signaling pathways, which are terminal signals in the unfolded protein response (UPR). The function of UPR is to detect protein misfolding to restore ER homeostasis under cellular perturbations^41^. Additionally, several studies have reported that ER, as an intracellular metabolic regulator, plays a pivotal role in endothelial dysfunction^42–44^. ER stress also acts as an active inflammatory trigger, accelerating the progression of endothelial dysfunction via inflammation and oxidative stress^45^. Besides, ER Ca^2+^ released from stressed cells increases the mtROS production and amplifies the activation of the NLRP3 inflammasome^46^. Accordingly, the mutual promotion between mitochondrial dysfunction and ER stress contributes to the activation of NLRP3 inflammasome and endothelial dysfunction, both of which are involved in oxidative stress and the production of ROS.

## Implications of NLRP3 inflammasome activation in endothelial dysfunction

### NLRP3 inflammasome activation in endothelial inflammation

**Fig. 4:**
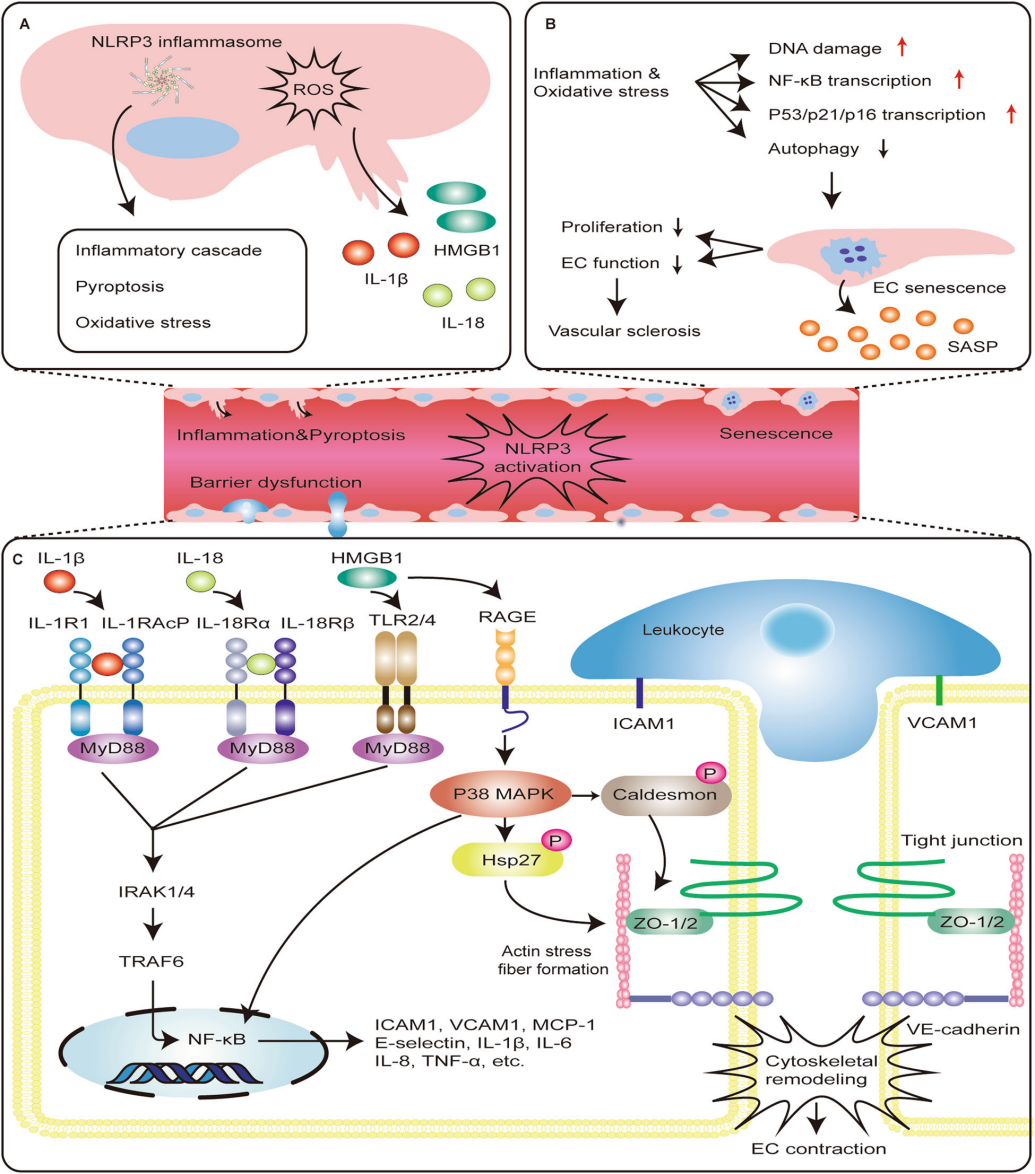
The role of NLRP3 inflammasome activation in endothelial dysfunction. **a** The secretion of mature forms of IL-1β, IL-18, and HMGB1 is the result of activation of NLRP3 inflammasome. These mediators possess properties of pro-inflammatory activation. Therefore, NLRP3 inflammasome can induce a potent inflammatory response, oxidative stress, and pro-inflammatory cell death called pyroptosis. **b** Inflammation and oxidative stress can induce endothelium DNA damage, activate NF-κB signaling pathway, increase P53/P21/P16 transcription, and inhibit autophagy, which may promote the process of endothelial cell senescence. Senescent cells can secrete senescence-associated secretory phenotype (SASP), including pro-inflammatory mediators, which may promote endothelial dysfunction and eventually lead to vascular sclerosis. **c** IL-1β binding to its cell surface receptor IL-1 receptor 1 (IL-1R1) recruits IL-1 receptor accessory protein (IL-1RacP) to activate intracellular signaling molecules, including myeloid differentiation factor 88 (MyD88), IL-1 receptor-associated kinase 1/4 (IRAK1/4), and TNF receptor-associated factor (TRAF), which then causes NF-κB activation. IL-18 binding to its cell surface receptor IL-18 receptor α chain (IL-18Rα) recruits IL-18 receptor β chain (IL-18Rβ) to activate similar intracellular signaling molecules. HMGB1 can also activate the NF-κB signaling pathway, which is downstream of toll-like receptor (TLR)2/4 activation. The activation of NF-κB signaling pathway increases the secretion of pro-inflammatory mediators such as cytokines and chemokines to mediate the adhesion of leukocyte and promote leukocyte extravasation. Additionally, the binding of HMGB1 to the RAGE receptor leads to the activation of downstream p38 MAP kinase, resulting in phosphorylation of the actin-binding protein Hsp27 and caldesmon, which causes actin stress fibers to form, cytoskeletal remodeling, and endothelial contraction. All of these reactions increase endothelial permeability by altering cell contractility and disrupting intercellular connections.

Generally, microbes, exogenous and endogenous crystals, and metabolic dysbiosis could trigger NLRP3 inflammasome activation, which initiates the secretion of mature forms of IL-1β and IL-18 from cells to promote further inflammatory processes and oxidative stress in the endothelium (Fig. 4)^47^. This intense inflammasome activation increases the release of new DAMPs, forming a negative-feedback loop^48^. The cytokine output of inflammasomes, specifically IL-1β, is a key inflammatory mediator in response to microbial invasion and tissue damage. EC is a target cell of IL-1β, and it also produces IL-1β during inflammation^49^. The activation of IL-1β can trigger the activation of secondary inflammatory mediators, such as IL-6 and C-reactive protein^50^. Moreover, IL-1β can also promote the secretion of adhesion molecules and chemokines in ECs, inducing a potent pro-inflammatory response^50^. Endothelial inflammation may initiate the occurrence and progression of endothelial dysfunction and promote one another in subsequent processes.

A growing number of agonists have been demonstrated to promote vascular inflammation by activating the NLRP3 inflammasome, including the endogenous medium. For example, visfatin has been demonstrated to promote endothelial inflammation as a pro-inflammatory adipokine^51^. Xia et al. found that NLRP3 inflammasome activation is an underlying cause of visfatin-induced endothelial inflammation^52^. This inflammatory response can cause endothelial dysfunction, initiating atherosclerosis during obesity. Moreover, visfatin has also been shown to increase neointimal formation both in vitro and in vivo, which is partly attributed to the formation and activation of NLRP3 inflammasome and IL-1β secretion^52^.

NLRP3 inflammasome activation is also involved in exogenous substance-mediated endothelial inflammation. Xia et al. demonstrated that NLRP3 inflammasome activation is involved in tetrachlorobenzoquinone (TCBQ)-induced endothelial inflammation^53^. TCBQ not only promotes the post-translational activation of NLRP3 and IL-1β secretion in HUVECs, but also downregulates the ubiquitination of NLRP3, which facilitates its activation, as an active metabolite of pentachlorophenol (PCP). This study also reported that K^+^ efflux, mtROS production, and mtDNA damage might contribute to TCBQ-induced NLRP3 inflammasome activation^53^. Further research revealed that GSDMD and mixed-lineage kinase domain-like (MLKL) play crucial roles in TCBQ-induced atypical inflammatory pathway activation^54^. The results showed that TCBQ aggravated NLRP3 inflammasome activation via the disruption of intracellular and extracellular ion homeostasis, which was caused by the leakage of GSDMD and MLKL and the release of cell contents^54^. Many agonists that target the NLRP3 inflammasome signaling pathway and facilitate endothelial dysfunction are listed in Table 1.

**Table 1:** Summary of agonists targeting NLRP3 inflammasome signaling pathway and facilitating endothelial dysfunction.

Agonist	Category	Effects and potential mechanisms	Disease	References
TMAO	Choline metabolite	•↑ Endothelial inflammation •↑ ROS–TXNIP–NLRP3 pathway •↓ eNOS expression •↓ NO roduction	AS	^124^
Visfatin	Adipokine	•↑ Endothelial inflammation •↑ Neointimal formation •↑ NLRP3 formation and activation	Obesity	^52^
Acetate, propionate	SCFAs	•↑ Endothelial inflammation •↑ Neointimal formation •↑ NLRP3 formation and activation	AS	^125^
TCBQ	Toxicant	•↑ Endothelial inflammation •↑ NLRP3 activation, IL-1β secretion •↑ NLRP3 deubiquitination •↑ GSDMD and MLKL leakage	/	^53,54^
LCWE	L. casei cell wall extract	•↑ Endothelial inflammation •↑ Lysosome membrane permeability, cathepsin B release •↑ NLRP3 formation and activation	Coronary arteritis	^126^
Heme	Iron porphyrins	•↑ Endothelial inflammation •↑ NLRP3 activation, IL-1β secretion	Hemolytic diseases	^127^
Endothelial GPR124	Orphan receptor	•↑ Atherosclerosis progression •↑ Nitrosative stress •↑ NLRP3 activation	AS	^128^
AGEs	Glycation products	•↑ Endothelial inflammation •↑ ROS/TXNIP/NLRP3 pathway •↓ EPC numbers, NO level, and eNOS expression	Chronic kidney disease	^129^
SREBP2	Transcription factor	•↑ Endothelial inflammation •↑ The production of toxic lipids, cholesterol crystals, and necrotic cells •↑ NLRP3 activation	AS	^130^
Adora2a	Adenosine receptor	•↑ Cerebral endothelial inflammation •↑ NLRP3 activation	Ischemic stroke	^131^
Prdx6-aiPLA2 activity	Enzyme	•↑ Endothelial inflammation •↑ Endothelial NOX2 activation •↑ ROS/NF-κB/NLRP3 pathway	ALI	^132^
PGE2	Prostaglandin	•↑ Endothelial inflammation, apoptosis •↑ NLRP3 activation •↑ Inflammatory chemokines	Diabetic retinopathy	^133^
Acrolein	Unsaturated aldehyde	•↑ Pyroptosis •↓ Cell migration •↓ ROS-dependent autophagy •↑ NLRP3 activation	AS	^57^
Cadmium	Metal toxicant	•↑ Endothelial inflammation, pyroptosis •↑ mtROS-mediated NLRP3 activation	CVD	^58^
Nicotine	Alkaloid	•↑ Endothelial inflammation, pyroptosis •↑ ROS production, NLRP3 activation	AS	^134^
		•↑ Endothelial barrier dysfunction •↑ Cathepsin B-dependent NLRP3 activation •↑ HMGB1 release		^69^

### NLRP3 inflammasome activation in endothelial cell death

Typically, cell death mainly includes apoptosis and necrosis; however, an emerging type of cell-intrinsic death has recently been found, termed pyroptosis^55^. Pyroptosis, an inflammatory programmed cell death process, is another functional outcome of NLRP3 inflammasome activation, which is mediated by the membrane pore-forming activity of the GSDMD N-terminal domain released by caspase-1 cleavage^56^. Pyroptosis is manifested by cell membrane rupture, cellular lysis, and release of pro-inflammatory contents, including IL-1β, IL-18, as well as high-mobility group box 1 (HMGB1), signifying the inflammatory nature of pyroptosis, and distinguishing pyroptosis from other forms of cell death^55^. NLRP3 inflammasome-mediated pyroptosis has been identified as a potential cause of EC death (Fig. 4).

Our previous study reported that NLRP3-mediated pyroptosis is involved in EC death caused by certain stimuli. Acrolein, an exogenous toxin, is produced in response to environmental pollution. Jiang et al. reported that acrolein-induced EC death is attributed to a decrease in ROS-dependent autophagy, which promotes NLRP3 inflammasome activation and pyroptosis in HUVECs. It is generally known that autophagy is a negative regulator of NLRP3 inflammasome activation. Consequently, NLRP3 inflammasome activation plays a crucial role in this process^57^. Chen et al. illustrated a new molecular mechanism of EC death and inflammation caused by cadmium (Cd), an independent risk factor for various cardiovascular diseases. Cd exposure induces EC death and inflammatory response via mitochondrial ROS-mediated NLRP3 activation and pyroptosis in HUVECs^58^.

A recent study reported that genetic copy number variations (CNVs) might be linked to the activation of NLRP3 inflammasome and endothelial pyroptosis. Chen et al. found that the human neutrophil peptide (HNP)-encoding gene (*DEFA1/DEFA3*) CNVs play specific roles in sepsis^59^. Interestingly, they reported that transgenic mice carrying more copies of the *DEFA1/DEFA3* suffer from more severe sepsis and mortality than those with low copy numbers of *DEFA1/DEFA3* and wild-type mice, which is caused by broader endothelial barrier dysfunction and EC pyroptosis. Also, in a mouse lung microvascular endothelial cell line model, it was reported that HNP-1 induces EC pyroptosis and endothelial barrier dysfunction by directly targeting the purinergic receptor P2X ligand-gated ion channel 7 (P2X7) and subsequently activating the canonical NLRP3/caspase-1 pathway. Functional blockade of HNP1–3 alleviates endothelial pyroptosis and protects the mice from sepsis^59^. This study provided a novel molecular mechanism for NLRP3 activation to mediate endothelial pyroptosis.

### NLRP3 inflammasome activation in endothelial barrier dysfunction

The vascular ECs cover the intima of blood vessels, forming a semipermeable barrier between circulating blood and extravascular matrix. This barrier maintains the transport of solutes, fluids, and cells by coordinating the opening and closing of cell junctions^60^. The endothelial barrier dysfunction is characterized by the loss of local contact between ECs, and extravasation of plasma proteins, cells, or solutes^61^. Sustained inflammatory activation of the endothelium initiates the secretion of various inflammatory mediators, including IL-1, tumor necrosis factor (TNF), vascular endothelial growth factor, histamine, and bradykinin, leading to the internalization of VE cadherin and disruption of inter-endothelial junctions^62^. Therefore, endothelial barrier dysfunction is considered to be an important mechanism underlying many diseases associated with inflammation, where inflammatory mediators activate diverse signaling pathways in the endothelium, resulting in the increased endothelial permeability and inappropriate loss of solutes and cells^62^.

Moreover, IL-1β, IL-18, and HMGB1 released by NLRP3 activation may activate NF-κB pathway, which in turn promotes the transcriptional activation of chemokines and adhesion molecules, increases leukocyte adhesion, and changes in cell permeability, thereby playing a crucial role in endothelial barrier dysfunction. IL-1β and IL-18 are products of NLRP3 inflammasome activation. As inflammatory mediators, they can enhance the expression of adhesion molecules and chemokines in the endothelium via binding to their cell surface receptors respectively, and activating the NF-κB pathway (Fig. 4)^63^. Endothelial NF-κB activation initiates transcriptional activation of numerous genes, including chemokines and adhesion molecules^64^. These reactions can increase the adhesion and rolling of leukocyte and promote leukocyte extravasation. Additionally, activated leukocytes can secrete cytokines that mediate persistent inflammatory responses, such as IL-1 or TNF, which can increase vascular endothelial permeability by altering cell contractility and disrupting intercellular connections^62^.

HMGB1 is closely related to NLRP3 inflammasome activation and endothelial barrier dysfunction (Fig. 4). NLRP3 inflammasome activation can also release DAMPs such as HMGB1 to the extracellular environment^65^. Extracellular release of HMGB1 may be caused by noncanonical mechanisms along with IL-1β and IL-18, or it may be a passive consequence of cell lysis during pyroptosis^66^. Extracellular HMGB1 can bind to its receptors, including toll-like receptor 2 (TLR2), TLR4, and RAGE, thereby inducing inflammation and repair responses^66^. Moreover, HMGB1 can induce endothelial barrier disruption by increasing the contractile activity and endothelial permeability. These effects are mediated by binding of HMGB1 to the RAGE receptor, leading to downstream p38 MAPK activation as well as actin-binding protein Hsp27 phosphorylation^67^. Interestingly, HMGB1 was also demonstrated to increase the secretion of pro-inflammatory mediators such as cytokines and chemokines via mediating NF-κB signaling pathway, at the downstream of TLR2 and TLR4 activation. This may indirectly cause endothelial barrier dysfunction. However, this needs to be thoroughly investigated in the future^67^.

Previous studies have unraveled that endothelial barrier dysfunction induced by certain risk factors is linked to NLRP3 inflammasome activation, which is achieved via the release of HMGB1. Chen et al. reported that the activation of NLRP3 inflammasome and subsequent release of HMGB1 are the underlying causes of intercellular junction fracture in mouse vascular ECs (MVECs) treated with high glucose (HG)^68^. In contrast, NLRP3 deficiency has a protective effect on intercellular junction interruption in the coronary arterial ECs of diabetic mice^68^. Zhang et al. revealed that NLRP3 inflammasome is involved in triggering nicotine-induced endothelial barrier dysfunction^69^. This study suggested that nicotine enhances NLRP3 inflammasome activation and causes the release of HMGB1, leading to the damage of inter-endothelial junction and hyperpermeability^69^.

### NLRP3 inflammasome activation in endothelial cells’ senescence

Tissue aging is often accompanied by chronic inflammation, and the same goes for endothelial senescence^70^. By inducing vascular structural and functional changes, EC senescence actively regulates aging-associated vascular dysfunction (Fig. 4)^71^. Specifically, the disruption of the cell cycle increases ROS and oxidative stress, vascular inflammation, causes impaired Ca^2+^ signaling, high serum uric acid levels, and activates renin–angiotensin–aldosterone system, which is closely related to the premature senescence of ECs^71^. Recently, NLRP3 inflammasome has been demonstrated to correlate mild systemic inflammation with age-related functional decline^72^. NLRP3 inflammasome can sense the accumulation of DAMPs during aging and mediate the pro-inflammatory cascade both inside and outside the brain, which is independent of the noncanonical caspase-11 pathway^72^. Inhibiting abnormal NLRP3 inflammasome activity during aging reduces age-associated innate immune activation, attenuates multiple age-related chronic diseases, and prolongs the health span, consistent with the previous results^72^. Furthermore, Youm et al. showed that NLRP3 inflammasome activation also causes age-related thymic involution and immunosenescence^73^.

A most recent study demonstrated that NLRP3 inflammasome-mediated IL-1β has a pathogenic role in multiple distinct ocular aging diseases^74^. Nevertheless, little is known about whether NLRP3 inflammasome contributes to EC senescence and the underlying molecular mechanisms. Yin et al. explored the roles and potential mechanisms of NLRP3 inflammasome in EC senescence^75^. It was found that NLRP3 inflammasome activation promotes bleomycin-induced EC senescence by increasing the IL-1β secretion. Additionally, secreted IL-1β also significantly upregulates the expression of the senescence-related marker p53/p21 protein. During these processes, ROS plays a key role in inducing TXNIP–NLRP3 interaction^75^. The molecular mechanism and role of NLRP3 inflammasome in endothelial senescence remain to be further studied.

Recently, some pieces of literature have reported that some beneficial substances can reduce the senescence of ECs, at least in part, by inhibiting NLRP3 activation. Sun et al. demonstrated that purple sweet potato color (PSPC), one type of flavonoid derived from purple sweet potato, ameliorates endothelium senescence by restraining ROS production and NLRP3 inflammasome activation, and then reduces atherosclerotic lesions in insulin-resistant mice^76^. Additionally, they further investigated the correlation between NLRP3 inflammasome and autophagy and the underlying mechanisms of EC senescence. It was found that PSPC inhibits endothelial senescence via enhancing autophagy and keeping inflammasome activation in check^77^. With the clarification of the molecular mechanism and role of NLRP3 inflammasome in EC aging in the future, we believe that more drugs will target the NLRP3 inflammasome signaling pathway to treat endothelial aging-related diseases.

## Noncoding RNAs (ncRNAs) targeting the NLRP3 inflammasome signaling pathway in endothelial dysfunction

ncRNAs are a broad spectrum of RNA molecules that have high transcriptional activity, and regulatory and structural functions, but do not encode proteins. ncRNAs, such as microRNAs (miRNAs), long noncoding RNAs (lncRNAs), and circular RNAs (circRNAs), are novel regulators of cardiovascular risk factors and cell functions^78–81^. Additionally, ncRNAs have been demonstrated to precisely control gene expression and have gradually become indispensable regulators of inflammation and immunity^82,83^. Several studies have provided evidence that ncRNAs may regulate endothelial function by mediating the NLRP3 inflammasome signaling pathway; these studies were summarized in this section (see Table 2).

**Table 2:** Summary of ncRNAs targeting NLRP3 inflammasome signaling pathway in endothelial dysfunction.

ncRNA	Target gene	Effect on NLRP3 activation	Functions	Disease/research context	References
miR-22	NLRP3	Down	•↓ EC apoptosis •↓ Pro-inflammatory cytokines •↑ Tube formation	Coronary atherosclerosis	^135^
miR-495	NLRP3	Down	•↓ EC inflammation •↓ EC apoptosis •↑ EC proliferation	Myocardial ischemia/reperfusion injury	^136^
miR-15a	FOXO1	Down	•↓ EC inflammation	Diabetic retinopathy	^137^
miR-126	HMGB1	Down	•↓ EC inflammation	Diabetic retinopathy	^138^
miR-590-3p	NLRP1, NOX4	Down	•↓ EC inflammation, pyroptosis	Diabetic retinopathy	^139^
miR-20a	TXNIP, TLR4	Down	•↓ EC inflammation •↓ ROS generation	Atherosclerosis	^140^
miR-30c-5p	FOXO3	Down	•↓ EC inflammation, pyroptosis	Atherosclerosis	^141^
miR-383-3p	IL1R2	Down	•↓ EC inflammation •↓ EC apoptosis •↑ Tube formation	Coronary atherosclerosis	^142^
miR-20b	TXNIP	Down	•↓ EC senescence	H_2_O_2_-induced EC senescence	^143^
miR-101-3p	Bim	Down	•↓ EC apoptosis	Serum deprivation-induced EC apoptosis	^144^
miR-200a-3p	Keap1, NLRP3	Up	•↑ EC inflammation	Sepsis-induced brain injury	^145^
miR-92a	SIRT1, KLF2, and KLF4	Up	•↑ Endothelial innate immunity •↓ NO bioavailability	Atherosclerosis	^146^
miR-125a-5p	TET2	Up	•↑ EC inflammation, pyroptosis	Atherosclerosis	^147^
LncRNA MALAT1	miR-22	Up	•↑ EC inflammation, pyroptosis	Atherosclerosis	^148^
LncRNA MEG3	miR-223	Up	•↑ EC pyroptosis	Atherosclerosis	^149^
LncRNA NEXN-AS1	/	Down	•↓ EC pyroptosis	Atherosclerosis	^94^
Hsa_circ_0068087	miR-197	Up	•↑ EC inflammation •↑ Tube formation	T2DM	^150^

**Fig. 5:**
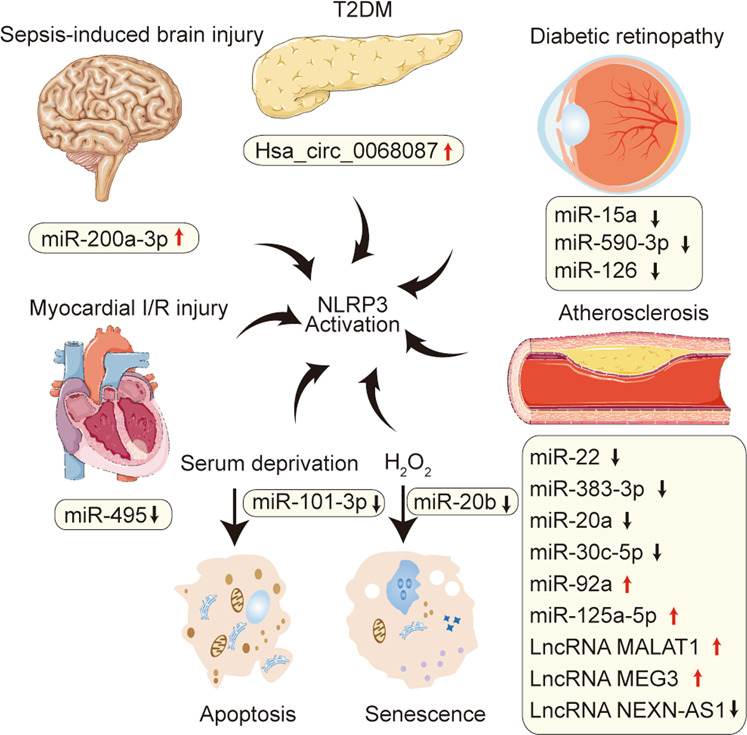
The involvement of noncoding RNAs (ncRNAs) in regulating NLRP3 inflammasome-mediated endothelial dysfunction and diseases. Schematic representation of ncRNAs upregulated or downregulated in multiple endothelial dysfunction-related disease models. These ncRNAs mediate endothelial dysfunction by directly or indirectly activating the NLRP3 inflammasome signaling pathway. The reported diseases mainly include myocardial ischemia/reperfusion (I/R) injury, sepsis-induced brain injury, type 2 diabetes mellitus (T2MD), diabetic retinopathy, and atherosclerosis. Moreover, miR-20b can be downregulated in H_2_O_2_-treated endothelial cells (ECs) and induces EC senescence by activating NLRP3 inflammasome. In addition, miR-101-3p is downregulated in serum deprivation-induced EC apoptosis, which is partially induced by activating the NLRP3 inflammasome signaling pathway. In this figure, the red upward arrow indicates upward adjustment, and the black downward arrow indicates downward adjustment.

Some exploratory research has demonstrated the potential of ncRNAs as clinical biomarkers or drug targets in various diseases^78,84–86^. Additionally, the distinctive roles of ncRNAs in the regulation of endothelial function have been widely explored^87–92^. Nevertheless, research on ncRNAs regulating endothelial function via the NLRP3 inflammasome signaling pathway is still limited (Fig. 5). ncRNAs tested in clinical samples have more potential as disease biomarkers in clinical applications. A great deal of clinical validation and mechanistic research is needed to elucidate the roles of ncRNAs regulating NLRP3 inflammasome signaling pathway in endothelial function.

## Clinical drugs and NLRP3 inflammasome and endothelial dysfunction-related diseases

### Statins

Statins are widely applied in the clinics because of their lipid-level-lowering properties. The functions of statins have been extended to include anticoagulant, anti-inflammatory, and immunomodulatory, suggesting that statins may have other benefits besides cholesterol reduction, as numerous clinical studies showed^93^. Wu et al. found that atorvastatin inhibited EC pyroptosis via increasing the expression of lncRNA NEXN-antisense RNA 1 (AS1) and its cognate gene NEXN, and inhibiting the expression of NLRP3 and pyroptosis-related molecules such as caspase-1 and IL-1β^94^. Simvastatin has been demonstrated to ameliorate endothelial dysfunction by inhibiting the activation of NLRP3 inflammasome in HG conditions. Mechanistically, simvastatin treatment remarkably enhanced the expression of zonula occluden-1 (ZO-1) and VE cadherin by inhibiting the NLRP3 inflammasome-dependent HMGB1 release, leading to the alleviation of vascular endothelial hyperpermeability^95^. Additionally, simvastatin can also exert an atheroprotective action via modulating the endothelial kruppel-like factor 2 (Klf2)-forkhead box P transcription factor 1 (Foxp1)–NLRP3 inflammasome network to suppress NLRP3 inflammasome activation^96^. These studies shed light on new mechanisms of statins to improve atherosclerosis and diabetic angiopathy by inhibiting NLRP3 inflammasome activation.

### Hypoglycemic agents

In recent years, studies have found that antidiabetic drugs can reduce endothelial disorders via blocking NLRP3 inflammasome activation, providing the potential for treating diabetic vasculopathy. The classification of these drugs based on pharmacological mechanisms is summarized as follows.

Dipeptidyl peptidase-4 (DPP-4) inhibitors are a class of effective hypoglycemic agents for the treatment of type 2 diabetes mellitus (T2DM). DPP-4 inhibitors have proven to be effective in improving endothelial function, reducing oxidative and pro-inflammatory states, suggesting beneficial effects on cardiovascular function. Qi et al. found that DPP-4 inhibitor vildagliptin ameliorates endothelial dysfunction, induced by high free fatty acid (FFA) levels through inhibiting the AMPK–NLRP3–HMGB1 pathway^97^. Moreover, vildagliptin also reduces the cellular lactate dehydrogenase (LDH) release and ROS levels, as well as protecting mitochondrial function and recovering eNOS levels, indicating its protective roles on endothelial dysfunction^97^. Recently, anagliptin, a novel DPP-4 inhibitor licensed for treating T2DM, was found to restore HG-induced endothelial dysfunction via SIRT1-dependent inhibition of NLRP3 inflammasome activation and suppression of NOX4–ROS–TXNIP–NLRP3 signaling, which suggests that anagliptin may have broad pharmacological effects in cardiovascular diseases and other metabolic disorders^98^.

Dulaglutide, a kind of glucagon-like peptide-1 receptor agonist (GLP-1 RA), has been approved for use in the treatment of T2DM. Luo et al. found that dulaglutide inhibits HG-induced endothelial dysfunction via SIRT1-dependent inhibition of NLRP3 inflammasome activation and expression of NOX4 and TXNIP^99^.

Acarbose, a well-known α-glucosidase inhibitor, is a postprandial acting antidiabetic drug. It has been reported that acarbose has protective effects against vascular endothelial dysfunction by inhibiting the production of NOX4 oxidase-dependent O_2_^•−^, which contributes to blocking NLRP3 inflammasome activation^100^. Furthermore, the reduced expression of NLRP3 inflammasome is also involved in the amelioration of vascular hyperpermeability by acarbose, which is attributed to the enhanced expression of junction protein ZO-1 and VE cadherin^100^.

Fenofibrate is a selective agonist of peroxisome proliferator-activated receptor α (PPARα) that prevents the progression of microvascular complications in T2DM. Deng et al. found that fenofibrate promotes wound healing via alleviating EPC dysfunction and stimulating angiogenesis in diabetic mice induced by streptozotocin (STZ), the effects of which are attributed to the inhibition of NLRP3 inflammasome pathway^101^.

Additionally, cilostazol, a phosphodiesterase-3 inhibitor, also alleviates the adverse vascular effects of living with diabetes. Cilostazol significantly reduces NLRP3 inflammasome activation and the activity of NOX4, TXNIP, HMGB1, IL-1β, and IL-18 in HAECs induced with FFA. Cilostazol also protected the function of SIRT1, which serves to limit the activity of NLRP3 inflammasome^102^.

Glibenclamide is a sulfonylurea drug widely prescribed to treat T2DM. Studies have discovered that glibenclamide attenuates blood–brain barrier (BBB) disruption in experimental intracerebral hemorrhage model by inhibiting NLRP3 inflammasome activation in microvessel ECs, thereby maintaining the integrity of the BBB^103^.

### Other anti-inflammatory or antioxidant drugs

Other anti-inflammatory or antioxidant drugs have also been investigated in different disease contexts. Aspirin is one of the most commonly used drugs for the secondary prevention of cardiovascular disease^104^. Zhou et al. found that aspirin protects the expression of ZO-1 and ZO-2 by inhibiting NLRP3 inflammasome formation and activation in LPS-induced MVECs and coronary arterial endothelium, thereby alleviating endothelial gap junction dysfunction^105^. This study unraveled a novel mechanism of aspirin in ameliorating endothelial dysfunction by blocking redox signaling and NLRP3 inflammasome activation, providing a new viewpoint on the clinical potential of aspirin in the early prevention of cardiovascular diseases^105^. The mitochondrion-targeting antioxidant mitoquinone (MitoQ) was found to inhibit endothelial inflammation and barrier injury in cigarette smoke extract (CSE)-treated HUVECs via restoring mitochondrial damage^106^. Mechanistically, the protective effects of MitoQ depend on suppressing the internalization of VE cadherin and cytoskeleton remodeling, and inhibiting the activation of NF-κB/NLRP3 inflammasome pathway, as well as restraining the production of mtROS and autophagy^106^.

**Fig. 6:**
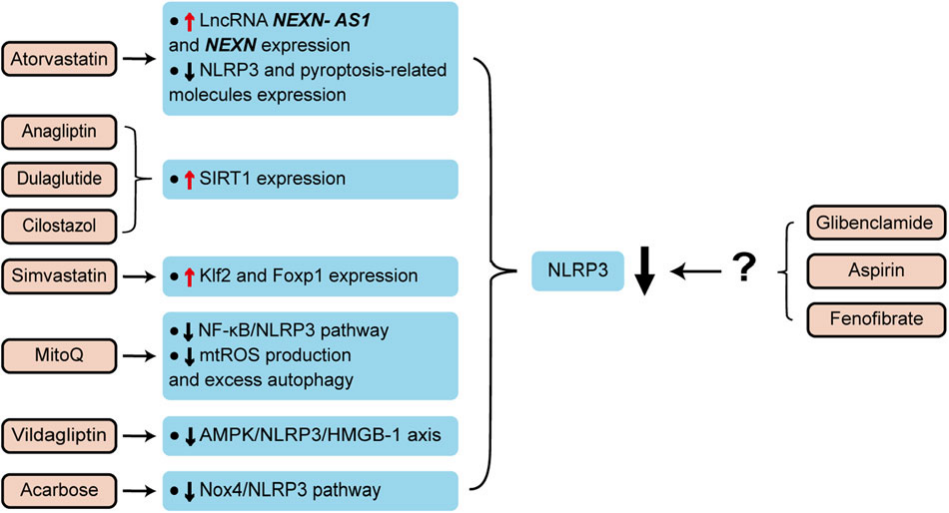
Drugs targeting NLRP3 inflammasome signaling pathway and their targets in endothelial dysfunction-related diseases. These drugs, including statins, hypoglycemic agents, and other anti-inflammatory or antioxidant drugs, ameliorate endothelial dysfunction by inhibiting NLRP3 inflammasome signaling pathway. The drugs on the left of the figure have been proved to inhibit NLRP3 inflammasome activation by mediating different targets, while the targets of the drug on the right of the figure still need further study to verify.

All these drugs ameliorate endothelial dysfunction by inhibiting NLRP3 inflammasome activation. Accordingly, we summarized the drugs targeting NLRP3 inflammasome signaling pathway and their targets in endothelial dysfunction-related diseases (Fig. 6). However, it is necessary to further study the molecular mechanism of these drugs inhibiting NLRP3 inflammasome pathway. Together, these studies provide new perspectives on the pharmacological mechanisms of drugs and therapeutic strategies for endothelial dysfunction-related diseases.

### NLRP3 inflammasome-specific pharmacological inhibitors for the treatment of endothelial dysfunction

NLRP3 inflammasome is involved in the progress of a variety of disorders, which promotes the discovery of NLRP3 inflammasome inhibitors. A large number of inhibitors have been demonstrated to improve endothelial dysfunction by inhibiting NLRP3 inflammasome signaling pathway, including endogenous and exogenous inhibitors, but with low specificity^107–109^. Here, we show the potential of specific NLRP3 inhibitors to ameliorate endothelial dysfunction (Table 3), which are briefly discussed below.

**Table 3:** Potential specific NLRP3 inflammasome inhibitors for the treatment of endothelial dysfunction.

Agents	Targets	Host	Functions	Disease	References
MCC950	ASC oligomerization	HRECs	•↑ Endothelial viability •↓ Endothelial apoptosis	Diabetic retinopathy	^112^
		BMVECs	•↓ BBB permeability •↑ Vascular integrity	Diabetes mellitus	^114^
		Diabetic mice	•↓ Vascular dysfunction •↑ ACh vasodilation	Diabetes mellitus	^113^
		CLP rat	•↑ Endothelial permeability	Sepsis	^115^
		HUVECs/CLP mice	•↑ Aortic vasodilation •↑ p-eNOS expression	Sepsis	^116^
Oridonin	Cysteine 279 of NLRP3	HUVECs	•↓ Vascular inflammation	Vascular inflammation	^119^
		HUVECs	•↓ Angiogenesis	Breast cancer	^120^
Tranilast	NLRP3 oligomerization	ApoE^−/−^ mice	•↓ Vascular inflammation •↓ Atherosclerosis	Atherosclerosis	^118^

MCC950 is one of the NLRP3 inflammasome-specific inhibitors by blocking ASC oligomerization and NLRP3 ATP hydrolysis^110,111^. A previous study reported that MCC950 treatment alleviated HG-induced human retinal endothelial cell (HREC) dysfunction probably in part by suppressing the NEK7–NLRP3 interaction^112^. Treatment of MCC950 also attenuated diabetes-related vascular dysfunction in diabetic mice model^113^. Moreover, treatment of diabetic rats after stroke with MCC950 ameliorated cognitive function, vascular permeability, and neurovascular remodeling^114^. More recently, it has been found that sepsis-related endothelial dysfunction was also improved with MCC950 intervention in in vivo and in vitro models^115,116^. Additionally, CY-09, OLT1177, 3,4-methylenedioxy-β-nitrostyrene (MNS), N-[3′,4′-dimethoxycinnamoyl]-anthranilic acid (Tranilast), and oridonin are also specific inhibitors of NLRP3 inflammasome^33,117^. Remarkably, a recent study found that tranilast exhibited antivascular inflammation and anti-atherosclerosis properties via increasing NLRP3 ubiquitination and impeding NLRP3 inflammasome activation^118^. Besides, oridonin was found to blunt endothelial inflammation through restraining the activation of MAPK and NF-κB signaling pathways^119^. It was also reported that oridonin diminished cell migration and angiogenesis in VEGF-treated HUVECs^120^.

Hence, NLRP3 inflammasome-specific pharmacological inhibitors may be the optimal choice for the treatment of endothelial dysfunction, providing a new strategy to treat endothelial dysfunction-related disease. In the future, the effects of these inhibitors are needed to be verified at the animal level and in clinical trials.

## Conclusions

In recent years, a group of evidence has accumulated linking NLRP3 inflammasome activation to the control of endothelial dysfunction. The findings reviewed here highlight the tremendous potential of direct or indirect modulation of NLRP3 inflammasome activation in combating various endothelial dysfunctions. When ECs are stimulated by exogenous substances or endogenous mediators, in addition to triggering oxidative stress, ER stress, mitochondrial dysfunction, and immune activation, these effects may also trigger a common signaling pathway of NLRP3 inflammasome activation, thereby exacerbating endothelial dysfunction. Of note, emerging literature indicates that certain well-known drugs, such as statins, hypoglycemic agents, and other anti-inflammatory or antioxidant drugs, can improve vascular dysfunction by inhibiting NLRP3 inflammasome signaling pathway. We have also summarized ncRNAs that involved in the regulation of endothelial function by directly or indirectly regulating NLRP3 inflammasome activation; this helps to understand the molecular mechanisms underlying NLRP3 inflammasome-related endothelial dysfunction, and may provide novel targets for the development of future therapeutics.

With the advancement of the research, our understanding of potential mechanisms that affect endothelial function through the NLRP3 inflammasome activation pathway is expanding. Meanwhile, the interactions between endothelial dysfunction and the NLRP3 inflammasome-regulated pathways may open up a new avenue for the treatment of cardiovascular diseases. While our understanding of the influence of NLRP3 inflammasome activation on endothelial dysfunction is continuously growing, our ideas on how NLRP3 inflammation perceives information about different mediators are still limited. Furthermore, it is unclear whether different agonists affect endothelial function through a single cascade of events or through distinct pathways to activate NLRP3 inflammation. In parallel, the effects of other inflammasomes, such as NLRP1, NLRC4, NLRP6, and AIM2, noncanonical inflammasomes, and upstream pathways on endothelial function, have not been thoroughly investigated so far.

Of the ncRNAs that regulate NLRP3 inflammasome pathway, miRNAs are currently the most studied. Accumulating research has indicated that miRNAs have tremendous potential as an indicative molecular biomarker or drug target in the diagnosis and treatment of diseases related to NLRP3 dysfunction. Nevertheless, plenty of thorny issues remain to be resolved to achieve this goal, including poor target specificity, poor miRNA stability, and the side effects involving liver damage^121^. Also, their effects may be restricted and removed by the kidney and liver due to the absence of protection around the carrier molecule^122^. Besides, the innate immune system may recognize and eliminate miRNA drugs^123^. However, it is encouraging that miRNA drug efficacy, delivery, and safety issues are currently being addressed. Shortly, miRNAs as therapeutic targets will be the focus of most clinical research. In the long run, as our understanding of other ncRNA mechanisms deepens, new ncRNAs may emerge as therapeutic targets. Additionally, the possible interaction between ncRNAs and NLRP3 inflammasome is only a preliminary study, and further research is needed in the future.

Excitingly, the expanding research on the role of NLRP3 inflammasome in endothelial dysfunction will hopefully enrich the understanding of the vital role of NLRP3 inflammasome in endothelial dysfunction-related inflammatory diseases. Furthermore, elucidating the molecular mechanisms of such interactions will be favorable for developing novel prevention approaches and more effective therapeutic strategies for diseases related to endothelial dysfunction, development of which is an exciting challenge and goal for future research.
